# A Follow-Up Study of Boys With Gender Identity Disorder

**DOI:** 10.3389/fpsyt.2021.632784

**Published:** 2021-03-29

**Authors:** Devita Singh, Susan J. Bradley, Kenneth J. Zucker

**Affiliations:** ^1^Department of Human Development and Applied Psychology, Ontario Institute for Studies in Education, University of Toronto, Toronto, ON, Canada; ^2^Department of Psychiatry, University of Toronto, Toronto, ON, Canada

**Keywords:** gender dysphoria, gender identity disorder, gender non-conformity, sexual orientation, DSM-5

## Abstract

This study reports follow-up data on the largest sample to date of boys clinic-referred for gender dysphoria (*n* = 139) with regard to gender identity and sexual orientation. In childhood, the boys were assessed at a mean age of 7.49 years (range, 3.33–12.99) at a mean year of 1989 and followed-up at a mean age of 20.58 years (range, 13.07–39.15) at a mean year of 2002. In childhood, 88 (63.3%) of the boys met the DSM-III, III-R, or IV criteria for gender identity disorder; the remaining 51 (36.7%) boys were subthreshold for the criteria. At follow-up, gender identity/dysphoria was assessed via multiple methods and the participants were classified as either persisters or desisters. Sexual orientation was ascertained for both fantasy and behavior and then dichotomized as either biphilic/androphilic or gynephilic. Of the 139 participants, 17 (12.2%) were classified as persisters and the remaining 122 (87.8%) were classified as desisters. Data on sexual orientation in fantasy were available for 129 participants: 82 (63.6%) were classified as biphilic/androphilic, 43 (33.3%) were classified as gynephilic, and 4 (3.1%) reported no sexual fantasies. For sexual orientation in behavior, data were available for 108 participants: 51 (47.2%) were classified as biphilic/androphilic, 29 (26.9%) were classified as gynephilic, and 28 (25.9%) reported no sexual behaviors. Multinomial logistic regression examined predictors of outcome for the biphilic/androphilic persisters and the gynephilic desisters, with the biphilic/androphilic desisters as the reference group. Compared to the reference group, the biphilic/androphilic persisters tended to be older at the time of the assessment in childhood, were from a lower social class background, and, on a dimensional composite of sex-typed behavior in childhood were more gender-variant. The biphilic/androphilic desisters were more gender-variant compared to the gynephilic desisters. Boys clinic-referred for gender identity concerns in childhood had a high rate of desistance and a high rate of a biphilic/androphilic sexual orientation. The implications of the data for current models of care for the treatment of gender dysphoria in children are discussed.

## Introduction

Gender identity is considered to be, for most people, a central aspect of one's sense of self (1–6).^1^ By around 3 years of age, if not earlier, most children can self-label themselves as either a boy or a girl (11–14) although cognitive-developmental gender theory suggests that the understanding of gender as an “invariant” aspect of the self does not occur until early to middle childhood, with the achievement of concreate operational thought (12, 15, 16). Gender differences in the adoption of gender role behavior, i.e., behavior associated with cultural definitions of masculinity and femininity, also emerge during the preschool years, if not earlier. These behaviors span various domains, including peer, toy, role play, and activity preferences [e.g., (3, 17, 18)]. Normative developmental research has long documented that, on average, both gender identity and gender role behaviors show significant and substantial between-sex differences (19–21). Later in development, sexual orientation also shows a substantial between-sex difference, i.e., most males are sexually attracted to females and most females are sexually attracted to males (19, 22).

In the 1950s and 1960s, a small clinical literature began to describe the phenomenology of children who displayed marked gender-variant behavior, including the strong desire to be of the other gender [e.g., (23–27)]. Subsequent volumes by Stoller (28) and Green (29) provided more comprehensive descriptions of such children. These early works were the sequel to the introduction of the diagnostic term Gender Identity Disorder (GID) of Childhood to the psychiatric nomenclature in the third edition of the *Diagnostic and Statistical Manual of Mental Disorders* [DSM-III; (30)], currently termed Gender Dysphoria (GD) in the DSM-5 (31). Since 1980, empirical research has examined a number of parameters pertaining to GID/GD: epidemiology, diagnostic and assessment methods, associated psychopathology, causal mechanisms, and therapeutic approaches [for reviews, see, e.g., (32–39)].

An additional parameter (the focus of the present study) pertains to the developmental course of GID in children. In the early literature, it was posited by some that pervasive gender-variant behavior in children might be a predictor of GID in adulthood (termed Transsexualism in the DSM-III) [e.g., (26, 40)]. At the same time, it was also recognized that gender-variant behavior in childhood was associated with sexual orientation (in males, androphilia, i.e., sexual attraction to men; in females, gynephilia, i.e., sexual attraction to women), but without co-occurring gender dysphoria [see, e.g., (41, 42); for a meta-analytic review, see (43)].

To date, there have been at least 10 follow-up studies of children whose behavior was consistent with the DSM diagnosis of GID (or GD per DSM-5) (44–53). Across these studies, the year at the time of first evaluation in childhood ranged from 1952 (49) to 2008 (51). For the 9 studies that included boys, the sample sizes (excluding those lost to follow-up) ranged from 6 to 79 (Mean age, 26 years). Most of these studies also provided the age at the time of first evaluation in childhood, which ranged from a mean of 7 years (47) to a mean of 9 years (48), with an age range from 4 to 12 years.

At the time of follow-up, using different metrics (e.g., clinical interview, maternal report, dimensional measurement of gender dysphoria, a DSM diagnosis of GID, etc.), these studies provided information on the percentage of boys who continued to have gender dysphoria (herein termed “persisters”) and the percentage of boys who did not (herein termed “desisters”).^2^ Of the 53 boys culled from the relatively small sample size studies (Bakwin, Davenport, Kosky, Lebovitz, Money and Russo, Zuger), the percentage classified as persisters was 9.4% (age range at follow-up, 13–30 years). In Green (47), the percentage of persisters was 2% (total *n* = 44; Mean age at follow-up, 19 years; range, 14–24); in Wallien and Cohen-Kettenis (52), the percentage of persisters was 20.3% (total *n* = 59; Mean age at follow-up, 19.4 years; range, 16–28); and in Steensma et al. (51), the percentage of persisters was 29.1% (total *n* = 79; Mean age at follow-up, 16.1 years; range, 15–19). Across all studies, the percentage of persisters was 17.4% (total *N* = 235), with a range from 0 to 29.1%.^3^

These studies also provided information on the sexual orientation of the boys at the time of follow-up. In the early studies, sexual orientation was ascertained from various sources (e.g., open-ended interviews with the patient, parent-report, chart information, etc.). In the more recent studies, sexual orientation was assessed in a more systematic manner, such as the use of a structured interview to assign a Kinsey-based rating of sexual orientation in fantasy and a rating of sexual orientation in behavior, dummy coded where a 0 = gynephilia and a 6 = androphilia [e.g., (47)].

Of the 53 boys culled from the relatively small sample size studies (op. cit.), 13 (34.2%) of the patients were classified as gynephilic and 25 (65.8%) were classified as biphilic/androphilic.^4^ In the remaining 15 patients (28.3% of the combined samples), their sexual orientation was either uncertain or unknown.

In Green's (47) study, 11 (25%) of the boys were classified as gynephilic (Kinsey ratings of 0–1) and 33 (75%) were classified as biphilic/androphilic in fantasy (Kinsey ratings of 2–6). For behavior, 6 (20%) were classified as gynephilic and 24 (80.0%) were classified as biphilic/androphilic. The remaining 14 boys (31.8% of the total sample) could not be classified with regard to behavior because they had had no interpersonal sexual experiences. In Green's study, the sexual orientation of a comparison group of boys, who had been recruited from the community, was also assessed: 100% of these boys (*n* = 35) were classified as gynephilic in fantasy and 96% (*n* = 25) were classified as gynephilic in behavior.

In the Wallien and Cohen-Kettenis (52) study, sexual orientation was assessed for attraction (2 items), fantasy (2 items), behavior (4 items), and sexual identity (1 item) using a self-developed Sexual Orientation Questionnaire. As in Green, Kinsey-type ratings were used in the analysis. Depending on the metric, data on sexual orientation were not available for anywhere between 22 and 40 (27.2–67.7%) patients. For attraction, 32% were classified as gynephilic and 68% were classified as androphilic (total *N* = 37); for fantasy, 19% were classified as gynephilic, 19% were classified as biphilic, and 62% were classified as androphilic (total *N* = 21); for behavior, 21% were classified as gynephilic, 16% were classified as biphilic, and 63% were classified as androphilic (total *N* = 19); lastly, for sexual identity, 19% were classified as gynephilic (“heterosexual”), 19% were classified as biphilic (“bisexual”), and 62% were classified as androphilic (“homosexual”) (total *N* = 27). Steensma et al. (51) used the same metrics as Wallien and Cohen-Kettenis. Depending on the metric, data on sexual orientation were not available for anywhere between 25 and 40 (31.6%-50.6%) patients. For attraction, 19.2% were classified as gynephilic, 15.4% were classified as biphilic, and 65.4% were classified as androphilic (total *N* = 52); for fantasy, 14% were classified as gynephilic, 22% were classified as biphilic, and 64% were classified as androphilic (total *N* = 50); for behavior, 35.9% were classified as gynephilic, 12.8 were classified as biphilic, and 51.3% were classified as androphilic (total *N* = 39); lastly, for sexual identity, 13% were classified as gynephilic (“heterosexual”), 27.8% were classified as biphilic (“bisexual”), and 59.3% were classified as androphilic (“homosexual”) (total *N* = 54).

In recent years, there have been various criticisms of these follow-up studies [see, e.g., (60–63); for a rebuttal, see (64)], particularly with regard to the putatively high percentage of desistance. It has been questioned, for example, to what extent the patients in these studies truly had GID/GD. For example, in the early studies, prior to the publication of DSM-III, one could reasonably argue that the diagnostic status of the patients was unclear because there were no formal diagnostic criteria to rely upon. However, one could argue in return that the behavior of these boys was phenomenologically consistent with the subsequent DSM criteria.

Consider, for example, the systematic study by Green [(47), Figure 1.2]. Green reported that 15% of the feminine boys, per parent-report, had “never” expressed the desire to be a girl or a woman at the time of the baseline assessment, 60% “occasionally” had such a desire, and only 25% had such a desire “frequently.” Thus, a conservative critic might argue that only the last group would have met one of the key indicators for the GID/GD diagnosis in the DSM.^5^ On the other hand, suppose a boy “occasionally” voiced the desire to be a girl over a period of several years. One might want to make the case that this would be consistent with the DSM descriptors of “persistently” or “repeatedly,” etc. Of course, one could debate what would genuinely count as “occasionally” (in Green's trichotomous metric, it would be anything more than “never” and less than “frequently”). In any case, it is probably reasonable to argue that, in Green's study, some boys were threshold and some boys were subthreshold for the equivalent of a DSM diagnosis. Given that in Green's study only one boy persisted with gender dysphoria at the time of follow-up, the threshold-subthreshold distinction would not really matter.

Studies that employed DSM criteria for GID/GD allow for a more formal examination of the “No True Scotsman” argument (https://en.wikipedia.org/wiki/No_true_Scotsman).

In the Wallien and Cohen-Kettenis (52) study, the DSM-III-R criteria were used to diagnose GID. Of the 12 persisters, all met the criteria for GID at the time of the baseline assessment; in contrast, only 68% of the 47 desisters met the criteria for GID; the remainder were deemed subthreshold for the diagnosis. Thus, in their study, the threshold-subthreshold distinction appears to have been an important one in predicting outcome; nonetheless, it should be noted that 68% of the desisters had been threshold for the diagnosis in childhood—perhaps a strong rebuttal to the No True Scotsman argument. In Steensma et al. (51), the DSM-IV-TR criteria were used. Of the 23 persisters, 21 (91.3%) met the criteria for GID; in contrast, only 22 (39.3%) of the 56 desisters were threshold for the diagnosis, suggesting an even more substantial difference in the threshold-subthreshold distinction than was found in Wallien and Cohen-Kettenis. Although the latter percentage was lower than what was found in Wallien and Cohen-Kettenis, that almost 40% of the desisters met the criteria for GID in childhood still argues in favor that the children were desisting from something.^6^

From Wallien and Cohen-Kettenis (52) and Steensma et al. (51), one predictor of outcome, therefore, was the distinction between being threshold or subthreshold for the GID diagnosis in childhood. Dimensional measures of gender-variant behavior have also proven useful. In both Wallien and Cohen-Kettenis and Steensma et al., dimensional measures of sex-typed behavior in childhood also significantly discriminated between the persisters and desisters, with the former group having, on average, more severe gender-variant behavior at the time of the childhood assessment. Steensma et al. found two other predictors of persistence: boys who were assessed at an older age and boys who had made either a partial or complete gender “social transition” [see (68–70)]. Of the 12 boys who had partially or completely transitioned prior to puberty, 10 (83.3%) were classified as persisters. In contrast, of the 67 boys who had not socially transitioned, only 13 (19.4%) were classified as persisters.

In the present study, we provide follow-up data with regard to both gender identity (persistence vs. desistance) and sexual orientation (gynephilia vs. biphilia/androphilia) on the largest sample of boys studied to date. Apart from providing percentage data on these two variables, which will be discussed in a comparative perspective in relation to the prior studies and the epidemiological literature, we also examine the predictors of outcome in relation to both demographic and sex-typed behavior measures (including whether or not the boys were threshold or subthreshold for GID) collected at the time of the baseline assessment in childhood.

## Method

### Participants

The participants were 139 boys (“birth-assigned males”)^7^ who, in childhood, had been referred to and then assessed in the Gender Identity Service, Child, Youth, and Family Program at the Centre for Addiction and Mental Health (CAMH) in Toronto, Ontario between 1975 and 2009 (Mean year of assessment, 1989.36) and were adolescents or adults at follow-up (Mean year at follow-up, 2002.35).^8^

Participants entered the follow-up study through two methods of recruitment. The majority of participants (77%) were recruited for research follow-up. There were two main waves of participant recruitment through research contact, from 1986 to 1993 (*n* = 32) and then from 2009 to 2011 (*n* = 71). During the period of data collection, 32 patients re-contacted the service for clinical reasons (eight for gender dysphoria, six for sexual orientation, and 18 for heterogeneous concerns) [for details, see (77), Appendix E]. They were informed about the opportunity to participate in the follow-up study and subsequently completed the study protocol. The majority of the patient-initiated participants had contacted the clinic between the two main waves of research recruitment. Thus, from 1994 to 2008, the participants who entered the study were primarily those who had contacted the service for clinical reasons.

In the early wave of follow-up, a lower-bound age for participation was set at 14 years, but by the mid-1990s this was changed to a lower-bound age of 16 years. In total, 110 (79.1%) participants were at least 16 years of age and 29 (20.9%) were younger than 16. Across the entire period of data collection, eligible participants, after review of the medical chart, were contacted at random (other than the participants who had returned to the service for clinical reasons). Due to lack of study resources and time constraints, contact with 162 other eligible participants was not attempted.

In total, 145 patients were approached about the follow-up study, either through research contact (*n* = 113) or following their clinical involvement with the Gender Identity Service (*n* = 32). Six patients declined, which yielded a participation rate of 95.9%. For those recruited for research purposes, initial contact, by telephone, letter or email, was first made with the parents because the patients were minors at the time of the childhood assessment and may have had no recollection of their clinic attendance. A total of 19 (14.3%) potential participants could not be reached/traced through previous addresses, registrars, and personal contacts.

Of the 139 participants, 110 were seen for a face-to-face assessment. For various reasons, the remaining 29 patients could not be seen for the face-to-face assessment (e.g., lived in another province or country, “too busy,” severe mental health issues). For some patients, they provided some information over the phone or information was provided by the parents; thus, for these patients, it was possible to obtain some follow-up data about their gender identity and sexual orientation.

The demographic characteristics of the participants, including their age at assessment in childhood and at the time of follow-up, are shown in Table 1. The GID diagnosis in childhood was based on the DSM-III (*n* = 53), DSM-III-R (*n* = 46), or DSM-IV (*n* = 40) criteria applicable at the time of assessment.^9^ A total of 88 (63.3%) boys met complete DSM criteria for GID in childhood. The remaining 51 (36.7%) boys were subthreshold for a DSM diagnosis, but all had some indicators of GID, and, based on the historical information provided during the assessment, some would have met the complete DSM criteria at some point in their lives prior to their assessment in childhood.^10^ The percentage who met the complete DSM criteria for GID did not differ significantly as a function of DSM edition, $\chi_{\left(2\right)}^{2}$ < 1.

**Table 1:** Demographic characteristics (*N* = 139).

**Characteristic**	***M***	***SD***	**Range**	**%**
**From childhood**				
Age (in years)	7.49	2.66	3.33–12.99	
Year of birth	1981.87	7.50	1966–1996	
Year of assessment	1989.36	7.50	1975–2004	
IQ^a^	105.93	15.47	69–138	
Social class^b^	40.74	15.15	8.0–66.0	
Marital status^c^				
Two-parent family				64.7
Other				35.3
Caucasian				84.9
**At follow-up**				
Age (in years)	20.58	5.22	13.07–39.15	
Year of follow-up	2002.35	9.08	1986–2011	
Follow-up interval (in years)^d^	12.88	6.07	2.77–29.29	
IQ^e^^,^^f^	105.88	16.03	65–138	

a *Full-Scale IQ was obtained with age-appropriate Wechsler intelligence scales*.

b *Hollingshead's (78) Four Factor Index of Social Status (absolute range, 8–66)*.

c *Other included the following family constellations: single parent, separated, divorced, living with relatives, or in the care of a child protection agency*.

d *Interval denotes the time between childhood assessment and follow-up assessment*.

e *Full Scale IQ estimated using four subtests: Vocabulary, Comprehension, Block Design, and Object Assembly*.

f *An IQ score was available only for participants who completed the face-to-face assessment. Of these, scores were not available for one participant*.

### Procedure

The majority of participants who completed the face-to-face assessment were evaluated on a single day. Three participants were seen twice. In these instances, the participants completed the self-report measures during their second visit as the complexity of their clinical presentation extended the duration of the assessment. Participants were provided a stipend for their participation in the follow-up assessment and reimbursement for travel expenses. For participants followed-up prior to 2009 (*n* = 68), the data were collected by the third author; for those followed-up between 2009 and 2011, the data were collected by the first author (*n* = 71). The study was approved by the Institutional Review Boards at the Clarke Institute of Psychiatry (subsequently the Centre for Addiction and Mental Health; Protocol #198/2008–2011) and the University of Toronto.

### Measures

Below, we describe the measures from assessment and follow-up of relevance for this article. A list of all measures used in the follow-up study can be found in Singh [(77), Table 4].

#### Childhood Assessment

##### Cognitive Functioning

Based on the child's age at the time of assessment, the appropriate version of the Wechsler Intelligence Scale for Children was administered (WPPSI-R or the WISC-R/WISC-III/WISC-IV). Full scale IQ scores were used to characterize level of cognitive functioning.

##### Behavioral and Emotional Problems

Parents completed the Child Behavior Checklist (CBCL), a measure of behavioral and emotional problems (79). Although not the focus of the present study, it is noted here because we used three CBCL indices (sum of all behavior problems and Internalizing and Externalizing *T* scores) as part of an internal validity analysis when comparing participants vs. non-participants (see Results).

##### Sex-Typed Behavior

Five child informant and two parent informant measures were used to assess the participants' sex-typed behavior in childhood: (1) Draw-a-Person [DAP] test (80); (2) a free-play task (81); (3) the Playmate and Playstyle Preferences Structured Interview (PPPSI) (82, 83); (4) sex-typed responses on the Rorschach test (84); (5) the Gender Identity Interview for Children (GIIC) (85–87); (6) the Gender Identity Questionnaire for Children (GIQC) (88–90); and (7) a measure of activity level/extraversion [(39); see also (91)]. These child and parent informant measures all have established discriminant validity, that is, they significantly differentiated the boys clinic-referred for gender identity concerns from control boys [for reviews, see (18, 92)]. A Childhood Sex-Typed Behavior Composite was subsequently computed for each participant (see below).

#### Follow-Up Assessment

##### Cognitive Functioning

Four subtests from the age-appropriate version of the Wechsler Intelligence Scales were administered (Vocabulary, Comprehension, Block Design, and Object Assembly). The standard scores from the subtests were averaged to form a prorated IQ score for cognitive functioning (93).

##### Concurrent Gender Identity

Concurrent gender identity was evaluated using a semi-structured interview and self-report questionnaires. During an audiotaped interview, each participant was asked to describe their current feelings about being a biological male. They were also asked to describe positive and negative aspects about their gender identity. For example, participants who reported a “male” gender identity were asked to describe positive and negative aspects of being male. The semi-structured interview also included questions based on the adolescent and adult GID criteria outlined in the DSM-III-R or DSM-IV (65, 94). Participants were asked to respond to these questions according to the last 12 months with *No, Sometimes*, or *Yes* [for details, see (77), Appendix G].

Two self-report measures were also used to assess current gender identity and gender dysphoria: (1) The Gender Identity/Gender Dysphoria Questionnaire for Adolescents and Adults (GIDYQ-AA) (95–97) or (2) the Gender Dysphoria/Identification questionnaire (GDIQ) (98). The GDIQ was developed prior to the GIDYQ-AA. As such, the GIDYQ-AA was introduced to the protocol subsequent to the GDIQ and, as a result, the more recent participants completed the GIDYQ-AA while earlier participants completed the GDIQ.

The male version of the GIDYQ-AA was completed. This 27-item questionnaire measures gender identity and gender dysphoria in adolescents or adults; participants over the age of 17 completed the adult version and younger participants completed the adolescent version. The adolescent and adult versions are identical except that, in the adult version, the words “man” and “woman” are used instead of “boy” and “girl.” Each item was rated on a 1–5 point response scale with verbal anchor points ranging from *Never* to *Always* based on a time frame of the past 12 months. Coding was such that a “lower” score signified more gender dysphoria. Item examples include the following: “In the past 12 months, have you felt unhappy about being a man?” and “In the past 12 months, have you had the wish or desire to be a woman?” Principal axis factor analysis identified a one-factor solution that accounted for 61.3% of the variance. All factor loadings were ≥0.30 (median, 0.86; range, 0.34–0.96). The GIDYQ-AA has strong evidence for discriminant validity and a high threshold for specificity (i.e., low false positive rate for non-GID individuals) [see (95, 96, 99–102)].

The GDIQ (98) contains 8 items pertaining to gender identity and gender dysphoria. Factor analysis identified two factors, accounting for 31.4 and 12.5% of the variance, respectively (all factor loadings ≥0.45). Factor 1 consisted of five items pertaining to gender dysphoria and Factor 2 consisted of three items pertaining to gender role identification. For the present study, only the questions for Factor 1 were used. Each item was rated on a 3-point or 5-point scale for the past 12 months (see Appendix 1 in Supplementary Material).

Participants were classified as having persistent gender dysphoria if their mean score on the GIDYQ-AA was ≤ 3.00, in line with sensitivity and specificity analyses from other data sets (95, 96). For participants who did not complete the GIDYQ-AA, the GDIQ was used. A participant was classified as a persister if two or more of the following five items on the GDIQ were endorsed: wish to have been born a girl (Item 1), wish to have surgery to change body (Item 2), feel more like a girl than a boy (Item 3), wonder if would be happier as a girl (Item 4), and somewhat or very dissatisfied with being a boy (Item 5).

Information regarding participants' gender identity/gender dysphoria was also obtained during the semi-structured clinical interview and, therefore, allowed for cross-validation of these questionnaire data. For those participants who did not complete the face-to-face interview, clinical information regarding gender identity/gender dysphoria was obtained through self- or parent-report or chart review. Across the entire sample, the GIDYQ-AA was used to classify persistence or desistence for 64 participants, the GDIQ for 42 participants, and interview/chart data/parent report for 33 cases.

##### Sexual Orientation

Sexual orientation in fantasy was assessed with specific questions from an audiotaped face-to-face interview and the self-report Erotic Response and Orientation Scale (EROS) (103).

The interview asked about four types of sexual fantasy over the past 12 months: (1) crushes on other people; (2) sexual arousal to visual stimuli (e.g., acquaintances, partners, and individuals from movies, television, etc.); (3) sexual content of night dreams; and (4) sexual content of masturbation fantasies. During the interview, participants were not asked directly about the gender of the person or persons who elicited sexual arousal, thus allowing time for the participant to provide this information spontaneously. Directed questions about the gender of the person(s) who elicited sexual arousal were asked only if the participant did not volunteer specific information about whether their arousal was directed to same-sex or opposite-sex individuals, or both. By the end of the interview, each participant provided information about sexual arousal to both same-sex and opposite-sex individuals. Using the Kinsey scale criteria (104), the interviewer assigned Kinsey ratings that ranged from 0 (exclusively gynephilic in fantasy) to 6 (exclusively androphilic in fantasy) for each question. A dummy score of 7 denoted that the participant did not experience or report any fantasies. A global fantasy score was also derived based on ratings from the four questions. Kinsey ratings for sexual orientation in fantasy were available for 129 participants.

Inter-rater reliability on Kinsey ratings for sexual orientation in fantasy was examined for 29 participants, selected at random. The second scorer listened to the audio recordings of the semi-structured interview, with specific attention to the information collected on sexual orientation. The inter-rater agreement on the Kinsey global fantasy rating was very good (kappa = 0.95) and the kappa values for the four specific components ranged from 0.81 to 1.00.

The EROS is a 16-item self-report measure assessing sexual orientation in fantasy over the past 12 months. Half of the questions pertained to gynephilic fantasy (e.g., “How often have you noticed that you had sexual feelings [even the slightest] while looking at a woman?”) and the other half pertained to androphilic fantasy (e.g., “How often have you noticed that you had sexual feelings [even the slightest] while looking at a man?”). Participants who were 18 years and older completed the adult version and younger participants completed the adolescent version. The adolescent and adult versions are identical except that, in the adult version, the words “man” and “woman” were used instead of “boy” and “girl.” Each item was rated on a 5-point scale for frequency of occurrence, ranging from 1 (“none”) to 5 (“almost every day”). Mean androphilic and gynephilic fantasy scores were derived for each participant. In the present study, we calculated a difference score between the participants' mean androphilic and gynephilic scores. Previous use of the EROS has shown good evidence of discriminant validity (98, 101).

Sexual orientation in behavior was assessed with specific questions during the face-to-face interview and with a modified version of the Sexual History Questionnaire (SHQ) (105). In the interview, questions asked about five types of sexual behavior: (1) dating; (2) holding hands in a romantic manner; (3) kissing; (4) genital fondling or touching a woman on the breasts, and (5) intercourse (penile-vaginal and anal). Kinsey ratings for behavior in the past 12 months were made in the same manner as fantasy ratings. Kinsey ratings for sexual orientation in behavior were available for 108 participants. Inter-rater reliability on Kinsey ratings for sexual orientation in behavior was examined for the same 29 participants. There was perfect inter-rater agreement on the Kinsey global behavior rating (kappa = 1.0) and the kappa values for the five specific components ranged from 0.91 to 1.00.

The modified SHQ consists of 20 questions. Ten questions pertained to gynephilic experiences (e.g., “How many women have you kissed on the lips in a romantic way?”) and 10 questions pertained to androphilic experiences (e.g., “How many men have you kissed on the lips in a romantic way?”). Participants who were 18 years and older completed the adult version and younger participants completed the adolescent version. The adolescent and adult versions are identical except that, in the adult version, the words “man” and “woman” were used instead of “boy” and “girl.” Each item was rated on a 5-point scale for frequency of occurrence, ranging from 1 (“none”) to 5 (“11 or more”), based on a time frame of the past 12 months. Mean total scores for gynephilic and androphilic experiences were derived. In the present study, we calculated a difference score between the participants' mean androphilic and gynephilic scores.

On the basis of Kinsey ratings, participants who completed the face-to-face interview were classified, similar to Green (47), into the following three sexual orientation groups for both fantasy and behavior: (1) gynephilic (Kinsey global ratings of 0–1); (2) biphilic/androphilic (Kinsey global ratings of 2–6), and (3) no sexual fantasy or behavior.

##### Social Desirability

Social desirability refers to the desire to cast a favorable impression on others. It can threaten the validity of self-report scales if in answering questions respondents seek social approval or try to represent themselves in a favorable manner (106). People scoring high on social desirability tend to provide socially acceptable answers regardless if their response accurately describes them. Participants 18 years and older completed the Marlow-Crowne Social Desirability Scale (M-CSDS) (107), which consists of 33 true-false items. The scale contains 18 culturally acceptable but unlikely statements keyed in the true direction and 15 socially undesirable but probable statements keyed in the false direction for a maximum possible score of 33. Participants 17 years and under were given a shorter version of the M-CSDS (108), containing 20 items that consist of 12 culturally acceptable but improbable statements keyed in the true direction and eight socially undesirable but probable statements keyed in the false direction for a maximum possible score of 20. For the present study, the percentage of endorsed socially desirable items was calculated for each participant. In order to integrate the data from both versions of the M-CSDS, participants' percentage score on each measure was converted to a proportion score which ranged from 0 to 1, which was used in all analyses. A higher proportion score indicates a greater propensity to give socially desirable responses. Several studies have found that the MCSDS is a reliable and valid measure of social desirability (107, 109, 110).

## Results

### Preliminary Analyses

#### Participants vs. Non-participants

Given that not all eligible participants were seen for follow-up, it is important to see to what extent the participants vs. non-participants were similar with regard to baseline characteristics, in part to gauge the internal validity of the sample (111).

The non-participants consisted of three subgroups: (1) patients who were eligible to participate in the study but were not contacted (*n* = 163), (2) patients who declined to participate (*n* = 6), and (3) patients who were not successfully traced (*n* = 19). Two sets of analyses were conducted to compare study participants vs. non-participants. First, the participants were compared to the patients who were eligible but not contacted. Second, the participants were compared to those who declined to participate and to those where contact was attempted but not successfully traced. Group comparisons were conducted on five demographic variables (age at assessment in childhood, IQ, ethnicity, and parents' marital status and social class), parent-report of behavior problems on the CBCL (three indices), and nine measures of childhood sex-typed behavior.

Of these 17 variables, there was only one significant difference between the 139 boys in the study compared to the 163 boys who were eligible to participate but were not contacted: participants had a higher IQ than non-participants, *t*_(289)_ = 2.01, *p* = 0.046.^11^ The effect size for this comparison was small (unpooled *d* = 0.22) [for details, see (77), Tables 5, 6]. When compared to the six cases where participation in the study was declined and to the 19 cases where the families could not be traced, there was also only one significant difference: parent's marital status, $\chi_{\left(2\right)}^{2}$ = 9.02, *p* = 0.011. The participants did not differ significantly from the non-participants who refused; however, they differed significantly from the cases that could not be traced, $\chi_{\left(1\right)}^{2}$ = 6.39, *p* = 0.012. The participants were more likely to have originated within a two-parent household than those who could not be traced. The comparison between the non-participants who refused and those who could not be traced approached significance (*p* = 0.056, Fisher's exact test). Again, the non-participants who could not be traced were more likely to have come from a family composition that was not two-parent. A further summary of comparisons between the participants and those who declined or could not be traced can be found in the Supplementary Material.

#### Participants: Method of Recruitment

Using *t*-tests or chi-square tests, the 107 participants who entered the study through research contact were compared to the 32 participants who were recruited into the study after they had re-contacted the clinic for clinical reasons on the demographic variables, CBCL behavior problems in childhood, and the measures of childhood sex-typed behavior. There were no significant differences between the two groups on the demographic variables of age at assessment, ethnicity or parents' social class and marital status (*p*s > 0.05). The comparison on childhood IQ approached significance, *t*_(137)_ = 1.97, *p* = 0.051, with the research entry participants having, on average, a higher IQ than the clinical entry participants. On the CBCL, there was a significant difference on Internalizing problems only, *t*_(137)_ = −2.02, *p* = 0.046, with the clinical entry participants rated by their parents as having more internalizing problems compared to the research entry participants. Of the nine measures of childhood sex-typed behavior, the two groups differed significantly on three: (1) free play, *t*_(119)_ = −2.11, *p* = 0.037, (2) the Gender Identity Interview for Children, *t*_(83)_ = −2.09, *p* = 0.04, and (3) the Gender Identity Questionnaire for Children, *t*_(95)_ = 2.39, *p* = 0.019, with the clinical entry participants having, on average, more childhood cross-gender behavior than the research entry participants. The percentage of clinical entry participants who were threshold for the diagnosis of GID in childhood did not differ significantly from the research entry participants (75.8 vs. 59.8%), $\chi_{\left(1\right)}^{2}$ = 1.83. Of the 32 clinical entry participants, 8 had re-contacted the clinic because of gender dysphoria. The above-described comparisons were repeated to compare the research and clinical entry participants but with these 8 participants excluded. With the eight participants who contacted the clinic for gender dysphoria removed, there were no significant group differences on demographic variables, CBCL behavior problems, and measures of childhood sex-typed behavior (all *p*s > 0.05).

### Gender Identity at Follow-Up

Appendix 2 in Supplementary Material shows the follow-up data for gender identity and sexual orientation for each participant. Of the 139 participants, 17 (12%) were classified as persisters and the remaining 122 (88%) were classified as desisters. The age at the time of follow-up did not differ significantly between the persisters (Mean, 20.12 years; SD = 5.54) and desisters (Mean, 20.64 years; SD = 5.19), *t*_(137)_ < 1. Of the 107 participants who, for research purposes only, were contacted for the follow-up study, 10 (9%) were classified as persisters; of the 32 participants who were recruited into the study after they were seen for some type of clinical concern, 7 (22%) were classified as persisters. The difference in persistence rate as a function of recruitment entry type was not significant, $\chi_{\left(1\right)}^{2}$ = 2.53, *p* = 0.112. The difference in persistence rate between those patients seen for the face-to-face assessment vs. those who were not (14.5 vs. 3.4%) was also not significant, $\chi_{\left(1\right)}^{2}$ = 1.70, *p* = 0.192. Supplementary Table 1 summarizes information on some domains of gender role outcome for the 17 participants classified as having persistent gender dysphoria.

For the 42 participants where the GDIQ was used to determine gender identity status at follow-up, four were classified as persisters and 38 were classified as desisters. Of the 38 desisters, three endorsed one item and the remainder endorsed none of the items.^12^ The four participants classified as persisters endorsed between three and five items.

For the 64 participants where the GIDYQ-AA was used to determine gender identity status at follow-up, 12 were classified as persisters and 52 were classified as desisters. All 52 desisters had a mean score >3.00 on the GIDYQ-AA. Of the 12 persisters, 10 had a mean score ≤ 3.00 and two had mean scores that were >3.00. In spite of having mean scores on the GIDYQ-AA that were above the recommended cutoff for caseness (95), these two participants were considered persisters because their clinical interview data indicated that they were experiencing significant gender dysphoria. Thus, clinical judgment was used to make the final classification for these two participants.

For the remaining 33 participants, clinical interview, parent-report or chart data were used to classify the percentage who were persisters (*n* = 1; 3%) or desisters (*n* = 32; 97%).

The persistence rate of gender dysphoria was examined as a function of participants' GID diagnostic status in childhood (threshold vs. subthreshold). Of the 88 participants who met the full diagnostic criteria for GID in childhood, 12 (13.6%) were classified as persisters and the remaining 76 (86.4%) were not. Of the 51 participants who were subthreshold for the GID diagnosis in childhood, 5 (9.8%) were classified as persisters and the remaining 46 (90.2%) were not. A chi-square analysis indicated that the rate of persistence did not differ significantly between the threshold and subthreshold groups, $\chi_{\left(1\right)}^{2}$ < 1.

Over the years, prevalence rates for gender dysphoria in adults have varied considerably. The variation is likely a function of many factors, including definition, time period, and source of ascertainment. For example, in the Standards of Care of the World Professional Association for Transgender Health (112), probably relying on an estimate given in the DSM-IV-TR, the prevalence of gender dysphoria in adult males was estimated to be 1 in 30,000. In the meta-analysis by Arcelus et al. (113), the prevalence in adult males was estimated at 1 in 14,705. Lastly, Zhang et al.'s (114) review of recent population-based surveys estimated the prevalence of a self-reported transgender identity in adults to range between 0.33 and 0.53% (males and females combined). Regardless of which base rate figure one might choose to use as a point of comparison, the persistence rate of 12% (while low in an absolute sense) would be considerably higher than what one would detect in the general population.

### Sexual Orientation at Follow-Up

Table 2 shows the Kinsey ratings for sexual orientation in fantasy. Data were not available for 10 participants, all of whom were desisters with regard to gender dysphoria. Based on the global rating for sexual orientation in fantasy, 43 (33.3%) participants were classified as gynephilic in fantasy and 82 (63.6%) were classified as biphilic/androphilic in fantasy. In the remaining four (3.1%) cases, the participants were classified as having no sexual fantasies and, therefore, a Kinsey rating could not be assigned.^13^ In all four cases, the participants were desisters. Of the 17 participants classified as persisters, 1 (5.9%) was gynephilic in fantasy and 16 (94.1%) were biphilic/androphilic in fantasy. For participants assigned a Kinsey rating between 0 and 6 in fantasy, we correlated the interviewer's Kinsey rating with the participants' responses on the EROS in which their mean gynephilic score was subtracted from their mean androphilic score. This yielded an *r*(101) = 0.86, *p* < 0.001.

**Table 2:** Kinsey ratings for sexual orientation in fantasy and behavior.

**Variable**	**Kinsey rating (fantasy)^a^**															
	**0**		**1**		**2**		**3**		**4**		**5**		**6**		**No fantasy**	
	***N***	**%**	***N***	**%**	***N***	**%**	***N***	**%**	***N***	**%**	***N***	**%**	***N***	**%**	***N***	**%**
Crush	36	36.7	0	0	2	2.0	4	4.1	2	2.0	11	11.2	29	29.6	14	14.3
Visual	31	31.6	1	1.0	2	2.0	10	10.2	3	3.1	12	12.2	29	29.6	10	10.2
Dreams	13	13.3	1	1.0	1	1.0	4	4.1	3	3.1	3	3.1	27	27.6	46	46.9
Masturbation	21	21.9	2	2.1	3	3.1	6	6.3	2	2.1	7	7.3	33	34.4	22	22.9
Global fantasy rating	40	31.0	3	2.3	3	2.3	8	6.2	2	1.6	14	10.9	55	42.6	4	3.1
	**Kinsey rating (behavior)^a^**															
	**0**		**1**		**2**		**3**		**4**		**5**		**6**		**No sexual behavior**	
	***N***	**%**	***N***	**%**	***N***	**%**	***N***	**%**	***N***	**%**	***N***	**%**	***N***	**%**	***N***	**%**
Holding hands	26	26.3	0	0	0	0	5	5.1	1	1.0	1	1.0	35	35.4	31	31.3
Kissing	21	21.2	0	0	0	0	6	6.1	2	2.0	2	2.0	34	24.3	34	34.3
Genital/breast contact	13	13.1	0	0	0	0	3	3.0	2	2.0	1	1.0	35	35.4	45	45.5
Intercourse	8	8.2	0	0	0	0	3	3.1	2	2.0	0	0	27	27.6	58	59.2
Global behavior rating	28	25.9	1	0.9	0	0	4	3.7	3	2.8	1	0.9	43	39.8	28	25.9

a *0 = Exclusively gynephilic to 6 = Exclusively androphilic*.

Table 2 also shows the Kinsey ratings for sexual orientation in behavior. Data were available for 108 participants. Based on the global rating for sexual orientation in behavior, 29 (26.9%) participants were classified as gynephilic and 51 (47.2%) were classified as biphilic/androphilic. The remaining 28 (25.9%) participants did not report any sexual behaviors in the 12 months preceding the follow-up assessment. For participants assigned a Kinsey rating between 0 and 6 in behavior, we correlated the interviewer's Kinsey rating with the participants' responses on the SHQ in which their mean gynephilic score was subtracted from their mean androphilic score. This yielded an *r*(75) = 0.79, *p* < 0.001.

For those participants who could be assigned a Kinsey rating (i.e., excluding those participants who did not report any sexual fantasies or behavior or for whom data were not available), the correlation between Kinsey global fantasy and global behavior ratings was very strong, *r*(78) = 0.92, *p* < 0.001.

### Group Classification as a Function of Gender Identity and Sexual Orientation in Fantasy at Follow-Up^14^

Combining gender identity (i.e., persister or desister) and sexual orientation in fantasy (i.e., gynephilic or biphilic/androphilic) at follow-up, the participants were classified into one of four outcome groups (for which we had all of the relevant data): (1) persistence of gender dysphoria with a biphilic/androphilic sexual orientation (*n* = 16); (2) desistance of gender dysphoria with a biphilic/androphilic sexual orientation (*n* = 66); (3) desistance of gender dysphoria with a gynephilic sexual orientation (*n* = 42); and (4) persistence of gender dysphoria with a gynephilic sexual orientation (*n* = 1). The participants who reported no sexual fantasies (*n* = 4) could not be included in this outcome classification. Given that only one participant was classified as gender dysphoric with a co-occurring gynephilic sexual orientation (Group 4), this category was excluded from subsequent analyses that compared these outcome groups.

#### Demographic Characteristics in Childhood as a Function of Gender Identity and Sexual Orientation in Fantasy

**Figure 1:**
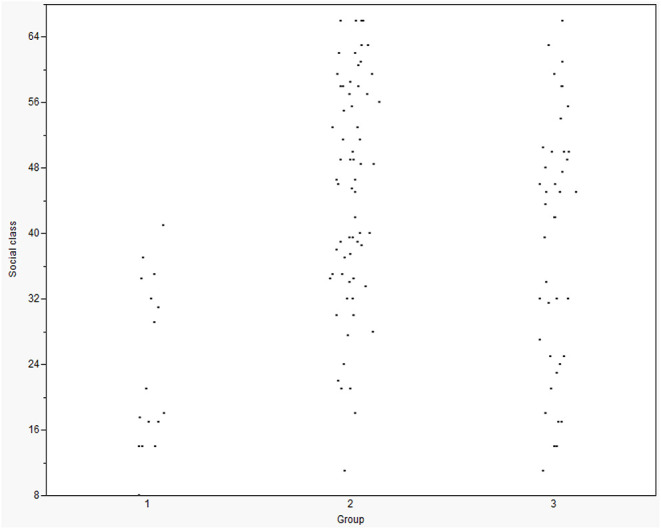
Distribution of social class for the outcome groups at follow-up. 1 = Biphilic/androphilic persisters (*n* = 16; *M* = 23.76, *SD* = 10.22). 2 = Biphilic/androphilic desisters (*n* = 66; *M* = 44.97, *SD* = 13.64). 3 = Gynephilic desisters (*n* = 42; *M* = 39.44, *SD* = 15.91).

Table 3 shows the demographic variables in childhood as a function of group. One-way ANOVAs and chi-square were conducted to evaluate whether the outcome groups differed on these variables. The groups differed significantly on four of the five childhood demographic variables. Duncan's multiple range test for unequal Ns showed that the biphilic/androphilic persisters were, on average, significantly older at the time of the childhood assessment than both the gynephilic desisters and the biphilic/androphilic desisters, who did not differ significantly from each other. The biphilic/androphilic desisters had, on average, a higher IQ than the biphilic/androphilic persisters but did not differ significantly from the gynephilic desisters. There was no significant difference in childhood IQ score between biphilic/androphilic persisters and gynephilic desisters. The biphilic/androphilic persisters were significantly more likely to come from a lower social class background compared to the gynephilic desisters and the biphilic/androphilic desisters, who did not differ significantly from each other (see also Figure 1). The biphilic/androphilic desisters were more likely to be living with both parents compared to the biphilic/androphilic persisters. There was no significant difference on marital status between the two desister groups.

**Table 3:** Demographic characteristics as a function of group.

**Variable**		**Group**			***F or χ^2^***	***p***	**η^2^ or Cramer's V**
		**Persisters** **Biphilic/** **Androphilic** **(*n* = 16)**	**Desisters** **Biphilic/** **Androphilic** **(*n* = 66)**	**Desisters Gynephilic** **(*n* = 42)**			
**Childhood**							
Age (in years)	*M*	8.85	6.96	7.49	3.57	0.031	0.06
	*SD*	1.67	2.69	2.62			
IQ^a^	*M*	101.63	110.20	103.18	3.77	0.026	0.06
	*SD*	14.81	14.56	15.16			
Social class^b^	*M*	23.76	44.97	39.44	15.30	<0.001	0.20
	*SD*	10.22	13.64	15.91			
**Marital status^c^**							
Two-parent	*N* (%)	7 (43.8)	49 (74.2)	24 (57.1)	6.74	0.034	0.23
Other	*N* (%)	9 (56.3)	17 (25.8)	18 (42.9)			
**Ethnicity**							
Caucasian	*N* (%)	14 (87.5)	58 (87.9)	32 (76.2)	2.77	0.250	0.14
Other	*N* (%)	2 (12.5)	8 (12.1)	10 (23.8)			
**Follow-up**							
Age at follow-up (in years)^d^	*M*	20.32	22.13	17.85	10.41	<0.001	0.15
	*SD*	5.67	4.97	3.95			
IQ at follow-up^a^^,^^e^^,^^f^	*M*	99.07	110.47	104.19	3.82	0.025	0.07
	*SD*	16.29	13.54	17.50			
Follow-up interval (in years)	*M*	11.47	15.17	10.36	9.63	<0.001	0.04
	*SD*	6.77	6.03	4.85			
Social desirability^g^	*M*	0.44	0.43	0.52	3.07	0.051	0.07
	*SD*	0.17	0.18	0.19			

a *Full-Scale IQ was obtained with age-appropriate Wechsler intelligence scales*.

b *Hollingshead's (78) Four Factor Index of Social Status (absolute range, 8–66)*.

c *Other included the following family constellations: single parent, separated, divorced, living with relatives, or in the care of a child protection agency*.

d *Interval denotes the time between childhood assessment and follow-up assessment*.

e *Full Scale IQ was estimated using four subtests: Vocabulary, Comprehension, Block Design, and Object Assembly*.

f *An IQ score was available only for participants who completed the face-to-face assessment*.

g *Absolute range, 0.00–1.00. Higher score indicates a greater propensity to give socially desirable responses. Age at follow-up, IQ at follow-up, social class, and parent's marital status were co-varied*.

The demographic variables from childhood on which the three groups differed–age at assessment, IQ, social class, and marital status–were significantly correlated (*r*s ranged from |0.32–0.58|) [see Table 12 in (77)]. To evaluate the predictive status of these variables on group outcome at follow-up, a multinomial logistic regression was performed. Table 4 shows the results. For these analyses, the biphilic/androphilic desisters served as the reference group. Each coefficient, *B*, represents the change in the log odds for Group for a 1-unit increase in the corresponding predictor, controlling for all other predictors in the model. The next column presents the standard error (SE) for each *B*. The Wald statistic was the quantity used to determine the significance level of each predictor variable. The quantity, *e*^*B*^, is the multiplicative change in the odds of being classified as a biphilic/androphilic persister (Model 1) or a gynephilic desister (Model 2) for a 1-unit increase in the corresponding predictor, and thus 100 × (*e*^*B*^ – 1) represents the percentage change in the odds ratio for a 1-unit increase in that predictor (115).

**Table 4:** Multinomial logistic regression of group outcome at follow-up.

**Predictor**	**Biphilic/Androphilic persisters**					**Gynephilic desisters**				
	***B***	**SE**	**Wald**	***p***	***e^***B***^***	***B***	**SE**	**Wald**	***p***	***e^***B***^***
Age at assessment	0.11	0.14	0.62	0.433	1.12	−0.02	0.09	0.03	0.856	0.98
IQ	0.02	0.03	0.85	0.358	1.02	−0.02	0.02	1.91	0.167	0.98
Social class	−0.14	0.04	13.66	<0.001	0.87	−0.01	0.02	0.13	0.716	0.99
Marital status	0.76	0.80	0.88	0.349	0.47	−0.43	0.52	0.70	0.402	1.54

*Reference group is the Biphilic/Androphilic Desisters. This group was chosen as the reference because it had the largest group size*.

It can be seen from Table 4 that only social class had a significant contribution to the prediction of group outcome at follow-up (see also Figure 1). The biphilic/androphilic persisters had a 13% increase in odds of coming from a lower social class background compared to the biphilic/androphilic desisters. However, social class did not predict outcome when the two desister groups were compared.

Table 3 also shows the variables of age, IQ, and social desirability scores at follow-up as a function of group. One-way ANOVAs revealed that both age and IQ differed significantly among the three groups (*p*s <0.01), but social desirability scores did not. Duncan's multiple range test for unequal Ns showed that the gynephilic desisters were, on average, younger than both the biphilic/androphilic persisters and the biphilic/androphilic desisters (both *p*s < 0.05), who did not differ significantly from each other. Regarding IQ at follow-up, the results were similar to those for IQ in childhood. The biphilic/androphilic desisters had, on average, a higher IQ than the biphilic/androphilic persisters (*p* < 0.05) but did not differ significantly from the gynephilic desisters. There was no significant difference in IQ between the biphilic/androphilic persisters and the gynephilic desisters.

#### Childhood Sex-Typed Behavior as a Function of Gender Identity and Sexual Orientation at Follow-Up

Supplementary Table 2 shows the means or percentage scores (for dichotomous measures) of the nine sex-typed measures obtained at the assessment in childhood as a function of the three outcome groups. ANCOVAs (with age at assessment, IQ, social class, and marital status covaried) or chi-square were used to examine whether the groups differed on any of these variables.^15^ There was a significant difference between the groups on four child-report measures (first drawn person on the Draw-a-Person, free play, Gender Identity Interview, and cross-sex peer preference on the Playmate and Play Style Preferences Structured Interview, and one parent-report measure (Gender Identity Questionnaire for Children). A statistical summary of these individual measures can be found in the Supplementary Text and the data are shown in Supplementary Table 2.

The childhood sex-typed behavior measures on which the groups differed were all significantly correlated (*r*s ranged from |0.30–0.76|) [reported in (77), Table 15].^16^ From these six measures (first drawn person on the Draw-a-Person, free play, Gender Identity Interview, cross-sex peer preference on the Playmate and Play Style Preferences Structured Interview, cross-sex toy preference on the Playmate and Play Style Preferences Structured Interview, and the Gender Identity Questionnaire for Children), a composite score of childhood sex-typed behavior was derived for each participant by taking the average of the six variables (each expressed as *z*-scores).^17^ A higher composite *z*-score indicates more cross-gender behavior at the assessment in childhood.

To evaluate the influence of childhood sex-typed behavior and demographic variables on group outcome at follow-up, a multinomial logistic regression was performed using the composite score and the demographic variables on which the groups differed–age at assessment, IQ, and social class–as predictor variables. It can be seen from Table 5 that both social class and the composite score of childhood sex-typed behavior were significant predictors of group outcome at follow-up in the first model, which compared the biphilic/androphilic persisters to the biphilic/androphilic desisters.

**Table 5:** Multinomial logistic regression predicting group outcome at follow-up.

**Predictor**	**Biphilic/Androphilic persisters**					**Gynephilic desisters**				
	***B***	**SE**	**Wald**	***p***	***e^***B***^***	***B***	**SE**	**Wald**	***p***	***e^***B***^***
Age at assessment	0.26	0.16	2.90	0.09	1.30	−0.14	0.11	1.55	0.21	0.87
IQ	0.02	0.03	0.58	0.45	1.02	−0.03	0.01	2.77	0.10	0.97
Social class	−0.12	0.03	12.28	<0.001	0.89	−0.01	0.01	0.51	0.47	0.99
Composite *z*-score	1.32	0.55	5.82	0.02	3.74	−0.66	0.31	4.38	0.04	0.52

*Reference group is the Biphilic/Androphilic Desisters. This group was chosen as the reference because it had the largest group size. A preliminary analysis with marital status included as a predictor variable showed that it did not have a significant effect and was, therefore, excluded in the final regression model. As suggested by Reviewer 3, per Benjamin et al. (116), for the “discovery of new effects,” p-values between 0.05 and 0.005 should be viewed as “suggestive” (i.e., informative, but cautiously interpreted), and p-values < 0.005 as “significant” (i.e., stronger evidence for the implausibility of a difference merely by chance)*.

The biphilic/androphilic persisters had a 274% increase in odds of having a higher composite score (i.e., more childhood cross-gender behavior) and an 11% reduction in the odds of coming from a higher social class compared to the biphilic/androphilic desisters. Age at childhood assessment and IQ did not have a significant effect on group outcome (both *p*s > 0.05). In the second model, which compared the gynephilic desisters to the biphilic/androphilic desisters, the only significant predictor of group outcome was the composite measures of sex-typed behavior. The biphilic/androphilic desisters had a 48% increase in odds of having a higher composite score compared to the gynephilic desisters.

## Discussion

### Methodological Issues

We were not able to recruit into the study all eligible patients; however, our analyses which compared the participants vs. the non-participants did not show any substantive or pervasive differences with regard to the baseline assessment characteristics, suggesting that the internal validity of the sample was not grossly compromised (111). The majority of follow-up participants were recruited for research purposes; however, a minority entered the study after having been seen in adolescence for some clinical issue. There was some evidence that the patients who were enrolled in the study after recontacting the clinic were, on average, more extreme in their gender-variant behavior in childhood; however, the percentage who were threshold for the GID diagnosis in childhood did not differ significantly between the two subgroups. Although the percentage of persisters was higher in the subgroup that had recontacted the clinic than the subgroup recruited for research purposes only (22% vs. 9%), the difference was also not statistically significant. If anything, the direction of the difference would suggest that the overall rate of persistence may have been slightly overestimated had we relied entirely on a “research-only” follow-up sample.

Another methodological issue is that we relied on different metrics to assess gender identity and gender dysphoria at follow-up. For example, we replaced the GDIQ with the GIDYQ-AA as we viewed the latter as a better measure; in some instances, we relied solely on interview data or information available in the patient's medical chart. However, we did not detect any substantive difference in the percentage of persisters across these different sources of information and thus do not believe that such method variance challenges the validity of the findings.

Although a minority of participants were seen on more than one occasion for follow-up, the majority were not. Thus, our results and interpretation of the follow-up data are largely limited to one “moment in time,” at a mean age of 20.58 years. It would, of course, be of value to have additional follow-up of the patients as they move further into adulthood in order to assess the stability (or lack thereof) of the data with regard to both gender identity and sexual orientation. In our own clinical experience, for example, we have observed that some of the patients seen during adolescence “fluctuated” between self-identifying as transgender and self-identifying as gay. Others have noted that a small number of apparent or presumed desisters during adolescence subsequently identified as transgender when seen at a later point in time (117).

### Summary of Key Findings

The present study provided follow-up data with regard to gender identity and sexual orientation in boys referred clinically for gender dysphoria. There were three key findings: (1) the persistence of gender dysphoria was relatively low (at 12%), but obviously higher than what one would expect from base rates in the general population; (2) the percentage who had a biphilic/androphilic sexual orientation was very high (in fantasy: 65.6% after excluding those who did not report any sexual fantasies; in behavior: 63.7% after excluding those who did not have any interpersonal sexual experiences), markedly higher than what one would expect from base rates in the general population; (3) we identified some predictors (from childhood) of long-term outcome when contrasting the persisters with a biphilic/androphilic sexual orientation with the desisters with a biphilic/androphilic sexual orientation and when contrasting the desisters with a biphilic/androphilic sexual orientation and the desisters with a gynephilic sexual orientation.

The 12% persistence rate was somewhat lower than the overall persistence rate of 17.4% from the prior follow-up studies of boys combined. When compared to the three most methodologically sound follow-up studies, the persistence rate was higher than the 2.2% rate found by Green (47), but lower than the 20.3% rate found by Wallien and Cohen-Kettenis (52) and the 29.1% rate found by Steensma et al. (51). There is one methodological caveat regarding the Steensma et al. study that is worth noting. In their study, the mean interval between assessment and follow-up was relatively short (7.21 years). The patients were eligible for follow-up if they were at least 15 years of age. Given the relatively short interval between the assessment in childhood and the follow-up assessment in adolescence, this meant that patients who had been assessed at younger ages in childhood would not have been old enough to participate in the follow-up assessment. Given that Steensma et al. found that (older) age at the time of the assessment in childhood was a significant predictor of persistence, it is conceivable that their persistence rate was an overestimate. Nonetheless, in the broadest sense, our data were quite consistent with the general finding from the prior follow-up studies that desistance from gender dysphoria is by far the more common outcome.

In our study, we did not find that persistence was more common among boys who were threshold for the diagnosis of GID when compared to the boys who were subthreshold (13.6% vs. 9.8%) although the pattern was in the same direction as that found by Wallien and Cohen-Kettenis (52) and Steensma et al. (51). We would, therefore, argue that the threshold-subthreshold distinction should not be abandoned in future follow-up studies although such studies might profit from using a symptom count of DSM indicators in addition to the dichotomous coding of the diagnosis as threshold vs. subthreshold. Consistent with both Wallien and Cohen-Kettenis and Steensma et al., our composite measure of sex-typed behavior in childhood was a significant predictor of outcome in that the patients classified as persisters with a biphilic/androphilic sexual orientation had more severe gender-variant behavior than the patients classified as desisters with a biphilic/androphilic sexual orientation; in addition, desisters with a biphilic/androphilic sexual orientation had more gender-variant behavior than the desisters with a gynephilic sexual orientation. Thus, dimensional measurement of gender identity and gender role behaviors from childhood provides added nuance in characterizing longer term trajectories with regard to both gender identity and sexual orientation.

With regard to sexual orientation at follow-up, the percentage of patients with a biphilic/androphilic sexual orientation in either fantasy or behavior was reasonably similar to those reported on in the prior follow-up studies which included standardized assessment measures (47, 51, 52). This finding also converges with three representative, general population prospective studies (118–120) and many retrospective studies (43) which document a significant association between patterns of gender-typed behavior in childhood and later sexual orientation.

The multinomial logistic regression analysis (Table 4) also showed a trend for the persisters with a biphilic/androphilic sexual orientation to be older at the time of the assessment in childhood compared to the desisters with a biphilic/androphilic sexual orientation; however, when the composite measure of sex-typed behavior in childhood was added to the equation (Table 5), age at assessment in childhood no longer showed such a trend [cf. Steensma et al. (51)]. In our smaller study of girls with GID (46), the persisters were, on average, 2.5 years older than the desisters at the time of the assessment in childhood (11.08 vs. 8.59 years) although the difference was not significant. It is our view that age at the time of a childhood assessment in relation to long-term outcome should continue to be examined in future follow-up studies.

Social class was a significant predictor of outcome: the persisters with a biphilic/androphilic sexual orientation were from a lower social class background compared to the desisters with a biphilic/androphilic sexual orientation (even after controlling for the other demographic variables). Why might this be the case? Because we had not made formal a priori predictions of outcome regarding any of our demographic variables, it is, of course, important to see whether or not it will be replicated in new follow-up studies. At present, our interpretation of the social class effect reflects on its relationship to other literatures.

One possibility pertains to the notion that acceptance of a gay or homosexual sexual identity is less in “working class” subculture (121). If this is, in fact, the case, it has been argued that transitioning from male to female—the so-called “homophobic” hypothesis with regard to gender dysphoria in adults (122)–would allow an androphilic sexual orientation to be more acceptable. Future studies would need to systematically examine whether boys with persistent GID first attempt to live as gay men before transitioning to the female gender role and whether or not this temporal sequence, when it occurs, is related to social class background.

In the present study, it could be hypothesized that the parents of persisters held less favorable views of androphilia (homosexuality) compared to the desisters and thus predisposed to persistence in order to “normalize” one's sexual orientation. However, this is simply a conjecture as parental attitudes toward homosexuality were not measured in the study sample. Indeed, none of the follow-up studies to date on boys with gender dysphoria have specifically examined attitudes toward homosexuality as a predictor of outcome.

Social class could also be a proxy for other explanatory factors. For example, in the present study, a lower social class background was significantly correlated with age at assessment in childhood (*r* = 0.44) and families where there had been a separation/divorce, etc. (*r* = 0.58). If one wanted to make the case that a later age at assessment might be associated with persistence (for a variety of reasons), perhaps social class is associated with a “delay” in seeking out an assessment and possible treatment (e.g., family stress, various other mental health challenges in the child and/or the family, etc.). In one study comparing the demographic characteristics of children vs. adolescents clinic-referred for gender dysphoria, it was found that the adolescents were more likely than the children to come from a lower social class background and from families in which there had been a separation/divorce, etc. (123).

### Clinical Implications

What clinical implications might be drawn from our data on the persistence and desistence rates of gender dysphoria in children? First, it should be recognized that the boys in the current study were seen during a period of time when treatment recommendations, if such were made, often aimed to reduce the gender dysphoria between the child's felt gender identity and biological sex. If one peruses the treatment literature, such recommendations were carried out using many therapeutic modalities: psychotherapy or psychoanalysis, behavior therapy, group therapy, parent-counseling, and interventions in the naturalistic environment, such as encouragement of same-sex peer relations [see, e.g., (124–126); for reviews, see (127, 128)].^18^ In our own sample, the kinds of treatments that the boys received, if any, were quite variable but it is beyond the scope of this article to describe them in general [however, for examples, see (136, 140, 141)]. It can, however, be said with certainty that the vast majority of boys were seen during a particular period of time when the therapeutic approach of recommending or supporting a gender social transition prior to puberty was not made. Indeed, in the current study, there was only one patient who had socially transitioned prior to puberty (at the suggestion and support of the professionals involved in this individual's care) and this particular patient was one of the persisters with a biphilic/androphilic sexual orientation. Second, it should also be recognized that, for the boys seen in the current study, none who were in late childhood and had (likely) entered puberty (Tanner Stage 2) had received puberty-blocking hormone treatment (GnRH analogs) to suppress somatic masculinization (142, 143) until sometime during adolescence.

In contrast, in recent years, it has become more common for some clinicians to recommend a gender social transition prior to puberty [e.g., (69, 144–147); for discussion, see (148–150)]. It has also become more common for parents to have already implemented a gender social transition on their own, without any formal input from a health professional (151). As argued by Zucker (64, 152), this is a very different type of psychosocial treatment designed to reduce gender dysphoria when compared to the other kinds of treatments noted above that have been recommended over the years.

The study by Steensma et al. (51), which found the highest rate of persistence, included some patients who had made a partial or complete gender social transition prior to puberty and this variable proved to be a unique predictor of persistence (see the Introduction). Rae et al. (153) recruited from a variety of community groups a sample of 85 markedly gender non-conforming children (Mean age, 7.5 years), none of whom had socially transitioned at a baseline assessment. At the time of follow-up, at a mean of 2.1 years later, 36 (42.3%) had socially transitioned and 49 (57.6%) had not. Using a composite of various metrics of gender identity and gender role behaviors, Rae et al. found that those who subsequently socially transitioned had more extreme gender-variant behavior at baseline than those who had not. Thus, this short-term follow-up study was consistent with the longer-term findings reported on by Wallien and Cohen-Kettenis (52), Steensma et al. (51), and the present study.

To date, however, there are no long-term follow-up studies of clinic-referred samples of children who had all socially transitioned prior to puberty. Future follow-up studies should be able to capture a much larger subgroup of such children and compared to those who have not with regard to long-term outcome with regard to persistence and desistance [e.g., (154)]. The persistence-desistance rates found in this study and the ones preceding it can be used as a comparative benchmark for samples in which a social transition took place prior to puberty.

## Data Availability Statement

The raw data supporting the conclusions of this article will be made available by the authors, without undue reservation.

## Ethics Statement

The research protocol was reviewed and approved by Clarke Institute of Psychiatry (subsequently the Centre for Addiction and Mental Health) and the University of Toronto. All participants who completed the face-to-face assessment gave written informed consent.

## Author Contributions

DS contributed to the conceptualization, data collection, data analysis, interpretation, and writing of the paper. SB contributed to the conceptualization and interpretation of the study. KZ contributed to the conceptualization, data collection, data analysis, interpretation, and writing of the paper. All authors contributed to the article and approved the submitted version.

## Conflict of Interest

The authors declare that the research was conducted in the absence of any commercial or financial relationships that could be construed as a potential conflict of interest. The reviewer RB declared a past co-authorship with one of the authors KZ to the handling Editor.

## Footnotes

^1^In one study, Turner and Brown (7) found that school-age children rarely mentioned their gender when providing open-ended self-descriptions; the most frequent descriptor pertained to activities and preferences. Turner and Brown suggested that it might be the case that gender is so central to one's self-concept that it “goes without saying” (p. 709). In contemporary times in the West, a very small number of parents choose to not “gender” their children (“theybies”) by not referring to them as boys or girls (and, at times, not even announcing to others the child's biological sex), dressing them in gender-neutral ways, etc. Little is known about the gender identity and gender role patterns of these children (8–10).

^2^The terms persistence and desistance have been used for a long time in clinical developmental psychiatry and psychology [e.g., (54)]. Zucker (55) was the first to apply these terms to describe the developmental psychosexual trajectories of children diagnosed with GID.

^3^The percentages provided here differ somewhat from other summary reviews [(39), pp. 285–286, (56, 57)] because we have excluded patients who were seen for the first time in adolescence [for this reason, data from Zuger (58) are also not included]. One other follow-up study was conducted by Nakamura (59). Unfortunately, this dissertation is not available for purchase at ProQuest (Ann Arbor, MI) and is only available for loan at the University of Essex library. Due to COVID-19 restrictions, it is currently inaccessible (K. Clarke, personal communication to G. Rieger, June 15, 2020). The director of the clinic at the time when the data were collected does not have a copy of the dissertation (D. Di Ceglie, personal communication, June 15, 2020).

^4^As pointed out by Reviewer 1, biphilic is a dubious neologism, combining Latin and Greek derivatives. Diphilic would be the more accurate derivative. However, introducing this term would probably confuse many readers, so we have retained the term biphilic (see https://en.wikipedia.org/wiki/Androphilia_and_gynephilia).

^5^The situation is compounded even further because in the DSM-IV, unlike in the DSM-III and DSM-III-R (65), the stated desire to be of the other gender was not a necessary criterion for the diagnosis [for the rationale, see (66), pp. 483–486]. In DSM-5, the desire to be of the other gender does not require explicit verbalization; the clinician is allowed leeway in drawing inferences based on other sources of information [see (67), pp. 904–905].

^6^In the follow-up study by Drummond et al. (46) of 25 girls from our clinic, the desistance rate was 88%. Of the 22 desisters, 13 (59.0%) met the DSM-III, III-R or IV criteria for GID. In Wallien and Cohen-Kettenis (52), of the 9 girls who desisted, 55.5% met the DSM-III-R criteria for GID. In Steensma et al. (51), of the 24 girls who desisted, 58.3% met the DSM-IV criteria for GID.

^7^Two reviewers asked why we chose to use the noun “boys” instead of the noun “males.” In our view, the question was reasonable but also a matter of semantics and taste. The third edition of *The Oxford Dictionary of Current English* (71) defines boy as “a male child...” Thus, we believe that the two words can be used synonymously. Males can refer to any age in the life-span whereas boys connote childhood. The participants in our study were coded as male at the time of their birth in the hospital delivery record, of which we had the actual birth records for the majority of the participants in the current study (72). As per Bouman et al. (73), one would say that the participants were “assigned male at birth” and then declared socially to be “boys” (74).

^8^The clinic was established in 1975 at the Clarke Institute of Psychiatry (75, 76), which became part of the CAMH in 1998.

^9^For boys seen prior to the publication of DSM-III in 1980, the draft criteria were used.

^10^In DSM-III, termed Atypical Gender Identity Disorder; in DSM-III-R and DSM-IV, termed Gender Identity Disorder Not Otherwise Specified.

^11^IQ data were not available for 11 of the 163 boys who were eligible for the study but were not contacted.

^12^By “endorsed,” we mean that the participants answered other than “never” on Items 1–4 or response options d-e for Item 5 (see Appendix 1 in Supplementary Material).

^13^For 104 participants, the Kinsey rating in fantasy was based on the information provided in the face-to-face interview. For 21 other participants, the Kinsey rating in fantasy was based on self-report (by telephone), information available in the participant's health record, or parent-report. Participants were assigned a Kinsey rating of 6 if the participant self-identified as “gay” or if the health record indicated that the patient was “homosexual” or gay, etc. Participants were assigned a Kinsey rating of 0 if the patient self-identified as “straight” or “heterosexual,” etc. A chi-square test showed that the percentage of participants who were classified as Kinsey 0–1 vs. 2–6 did not differ significantly as a function sexual orientation ascertainment method, $\chi_{\left(1\right)}^{2}$ = 1.49.

^14^Given the strong correlation between Kinsey fantasy and behavior ratings and that there were fewer missing data on the Kinsey fantasy variable, participants were classified into one of the four outcome groups based on their fantasy ratings.

^15^The ANCOVA model was adjusted to accommodate a categorical covariate.

^16^Although the groups did not differ significantly on cross-sex toy preference on the PPPSI, this measure is included here because there was a trend in the direction of a significant group difference.

^17^For some participants, data were not available on all six measures. In these cases, the composite score was the average of the number of variables for which there were data.

^18^This “broad stroke” summary of therapeutic goals is not meant to minimize the complexity of ethical issues regarding how treatment has been conceptualized over the years [see, e.g., (129–133)]. In the early years, treatment recommendations included other goals: for example, Bakwin (44) wrote that “Suggestions for management…[were]…designed to encourage gender appropriate behavior *and* to prevent homosexuality” [p. 620, emphasis added; see also (134)]. Rekers (135) was subsequently quite transparent regarding the influence of his own religious beliefs in formulating treatment goals, sometimes congruent with parents' religious beliefs (see p. 131). Prayer appears to have guided Rekers' selection of behavior therapy as a treatment modality for the treatment of his patients with childhood GID (p. 131). Money and Russo (50) wondered what the course of psychosexual differentiation might be if “a group of boys with discordance of gender identity/role [were] transferred from the home of origin to, say, a children's recovery center or foster home…as happens in the case of child-abuse dwarfism…” (p. 40). In our own clinic, although some parents might have desired or requested that treatment be designed in order to prevent homosexuality, this was a goal that we never endorsed [see (136), pp. 391–393]. Over the years, many secular-minded clinicians–although clearly opposed to any type of preventive efforts with regard to sexual orientation–argued in favor of reducing gender dysphoria vis-à-vis natal sex, if that was feasible. Meyer-Bahlburg (125), for example, wrote: “…we cannot rule out the possibility that early successful treatment of childhood GID will diminish the role of a continuation of GID into adulthood. If so, successful treatment would also reduce the need for the long and difficult process of sex reassignment which includes hormonal and surgical procedures with substantial medical risks and complications” (p. 362). Along similar lines, Cohen-Kettenis and Pfäfflin (33) remarked: “Relatively little dispute exists regarding the prevention of transsexualism, though evidence about the effectiveness of treatment in preventing adult transsexualism is also virtually nonexistent” (p. 120). In more recent years, what the best-practice should be for the treatment of gender dysphoria in children has been widely discussed and debated, which highlight the various limitations of treatment effectiveness studies (137–139).
