# Supplementary material for: A Follow-Up Study of Boys With Gender Identity Disorder
Source: Front Psychiatry. 2021 Mar 29;12:632784. doi: 10.3389/fpsyt.2021.632784 (PMC8039393; doi:10.3389/fpsyt.2021.632784)
Supplement: Supplementary file 1 [file Table_1.doc]

| Supplemental Table 1 | | | | | | |
| --- | --- | --- | --- | --- | --- | --- |
| *Summary of Gender Dysphoric Participants* | | | | | | |
| ID | Age at follow-up  (in years) | Legal name change | Using female name | Living in female role | On puberty blockers and/or  cross-sex hormones | Any surgery |
| 1 | 18.24 | No | No | Androgynous | No | No |
| 2 | 19.38 | No | Yes | Androgynous | No | No |
| 3 | 35.14 | Yes | Yes | Yes | Yes | No |
| 4 | 19.08 | No | Yes | Yes | No | No |
| 5 | 26.04 | No | Yes | Yes | Yes | No |
| 6 | 18.82 | No | No | No data | No | No |
| 7 | 15.47 | No | No | No | No | No |
| 8 | 23.55 | Yes | Yes | Yes | Yes | No |
| 9 | 22.32 | No^a^ | Yes | Yes | Yes | No |
| 10 | 13.62 | No | No | No | No | No |
| 11 | 19.77 | No | Yes | Yes | Yes | No |
| 12 | 16.90 | No | No | No | No | No |
| 13 | 22.18 | No | Yes | Partially | No^b^ | No |
| 14 | 17.68 | No | Yes | Yes | Yes | No |
| 15 | 16.61 | No | No | No | No | No |
| 16 | 15.97 | No | Yes | Yes | Yes | No |
| 17 | 15.69 | No | Yes | Yes | Yes | No |
| ^a^This participant was in the process of applying for a legal name change.  ^b^Puberty suppressing hormones (blockers) were recommended. | | | | | | |
