# Supplementary material for: A Follow-Up Study of Boys With Gender Identity Disorder
Source: Front Psychiatry. 2021 Mar 29;12:632784. doi: 10.3389/fpsyt.2021.632784 (PMC8039393; doi:10.3389/fpsyt.2021.632784)
Supplement: Supplementary file 2 [file Table_2.docx]

| Supplemental Table 2 | | | | | | | | |
| --- | --- | --- | --- | --- | --- | --- | --- | --- |
| *Childhood Sex-Typed Behavior as a Function of Group* | | | | | | | | |
| Variable |  | Persisters  Biphilic/  Androphilic | | Desisters  Biphilic/  Androphilic | Desisters  Gynephilic | *F or χ^2^* | *p* | η^2^ or Cramer’s V |
| DSM Diagnosis^a^ |  |  | |  |  |  |  |  |
| Threshold for GID | N (%) | 12 (75.0) | | 46 (69.7) | 23 (54.8) | 3.29 | .193 | .16 |
| Subthreshold for GID |  | 4 (25.0) | | 20 (30.3) | 19 (45.2) |  |  |  |
|  |  |  | |  |  |  |  |  |
| Child-report measures | | | | | | | | |
| Draw-a-Person |  |  | |  |  |  |  |  |
| Same-Sex Person Drawn First | N (%) | 4 (25.0) | | 27 (40.9) | 25 (59.5) | 6.61 | .037 | .23 |
| Cross-Sex Person Drawn First |  | 12 (75.0) | | 39 (59.1) | 17 (40.5) |  |  |  |
| Free Play Task^b^ | M | .24 | | .23 | -.06 | 7.82 | .001 | .13 |
|  | SD | .43 | | .62 | .69 |  |  |  |
|  | N | 13 | | 60 | 37 |  |  |  |
| Rorschach Difference Score^c^ | M | .40 | | .97 | -.08 | .53 | .589 | .01 |
|  | SD | 4.75 | | 4.07 | 3.11 |  |  |  |
|  | N | 15 | | 61 | 36 |  |  |  |
|  |  |  | |  |  |  |  |  |
| Gender Identity Interview^d^ | M | 9.78 | | 5.91 | 7.43 | 4.24 | .018 | .11 |
|  | SD | 5.63 | | 5.31 | 7.10 |  |  |  |
|  | N | 9 | | 46 | 21 |  |  |  |
|  |  |  | |  |  |  |  |  |
| Playmate and Playstyle Preferences | M | 9.75 | | 8.13 | 8.08 | 3.54 | .042 | .20 |
| Structured Interview: Cross-sex Peer Play^e^ | SD | 4.83 | | 5.78 | 5.57 |  |  |  |
|  | N | 8 | | 16 | 12 |  |  |  |
|  |  |  | |  |  |  |  |  |
| Playmate and Playstyle Preferences | M | 8.75 | | 7.00 | 6.67 | 2.55 | .096 | .15 |
| Structured Interview: Cross-sex toy play^f^ | SD | 2.43 | | 2.92 | 3.37 |  |  |  |
|  | N | 8 | | 16 | 12 |  |  |  |
|  | | | | | | | | |
| Parent-report measures | | | | | | | | |
| Gender Identity Questionnaire for | M | | 2.40 | 2.88 | 2.87 | 4.05 | .021 | .09 |
| Children^g^ | SD | | .49 | .58 | .85 |  |  |  |
|  | N | | 10 | 53 | 22 |  |  |  |
|  |  | |  |  |  |  |  |  |
| Temperament: Activity Level^h^ | M | | 2.95 | 3.08 | 3.03 | .593 | .554 | .010 |
|  | SD | | .41 | .49 | .59 |  |  |  |
|  | N | | 15 | 65 | 41 |  |  |  |
| ^a^For diagnosis, GID means that the patient met complete DSM-III, DSM-IIIR, or DSM-IV criteria for GID. For the continuous measures of sex-typed play, age, IQ, social class, and parent’s marital status were co-varied.  ^b^Difference between cross-sex and same-sex play during a free-play task. Positive score indicates more cross-sex play.  ^c^Difference between number of cross-sex and same-sex responses. Positive score indicates more cross-sex responses.  ^d^Absolute range, 0-24. A higher score reflects more gender identity confusion.  ^e^Absolute range, 0-14. A higher score indicates more cross-sex playmate/peer choices.  ^f^Absolute range, 0-14. A higher score indicates more cross-sex toy choices.  ^g^Absolute range, 1-5. A lower score indicates more cross-gender behavior.  ^h^Absolute range, 1-5. A higher score indicates higher activity level. | | | | | | | | |
