# Supplementary material for: A Follow-Up Study of Boys With Gender Identity Disorder
Source: Front Psychiatry. 2021 Mar 29;12:632784. doi: 10.3389/fpsyt.2021.632784 (PMC8039393; doi:10.3389/fpsyt.2021.632784)
Supplement: Supplementary file 3 [file Data_Sheet_1.docx]

Supplemental Appendix 1

Gender Dysphoria/Identification Questionnaire (Factor 1)

1. In the past 12 months, how often have you wished that you had been born a girl instead of a boy?

a. never

b. sometimes

c. a lot

2. In the past 12 months, how often have you wished to have an operation to change your body into a girl’s?

a. never

b. sometimes

c. a lot

3. In the past 12 months, how often have you felt more like a girl than like a boy?

a. never

b. occasionally

c. a lot

4. In the past 12 months, how often have you wondered whether you would be happier as a girl than as a boy?

a. never

b. sometimes

c. a lot

5. In the past 12 months, how have you felt about being a boy?

a. very satisfied

b. somewhat satisfied

c. sometimes satisfied, sometimes dissatisfied

d. somewhat dissatisfied

e. very dissatisfied
