# Supplementary material for: A Follow-Up Study of Boys With Gender Identity Disorder
Source: Front Psychiatry. 2021 Mar 29;12:632784. doi: 10.3389/fpsyt.2021.632784 (PMC8039393; doi:10.3389/fpsyt.2021.632784)
Supplement: Supplementary file 4 [file Data_Sheet_2.doc]

Appendix 2

*Gender Identity and Sexual Orientation at Follow-up*

| **No.** | **Age at Assessment (in years)** | **Age at follow-up (in years)** | **Gender Identity** | **Kinsey Ratings** | | | **DSM** |
| --- | --- | --- | --- | --- | --- | --- | --- |
|  |  |  |  | **Fantasy** | **Behavior** | |  |
|  | 6.42 | 18.24 | Persister | 6 | 6 | | + |
|  | 5.69 | 15.05 | Desister | 0 | 0 | | + |
|  | 8.23 | 17.73 | Desister | 0 | 0 | | - |
|  | 6.39 | 15.15  18.27  39.15 | Desister | 6 | 6 | | + |
|  | 5.87 | 14.06  15.11 | Desister | 0 | 7 | | + |
|  | 5.42 | 14.15  16.26 | Desister | 7 | 7 | | + |
|  | 9.53 | 17.65 | Desister | 0 | 7 | | - |
|  | 5.07 | 13.96  15.39 | Desister | 0 | 7 | | + |
|  | 7.95 | 16.10 | Desister | 0 | 0 | | **-** |
|  | 12.85 | 20.14 | Desister | 5 | 6 | | - |
|  | 5.33 | 14.15  16.80  20.23 | Desister | 5 | 7 | | + |
|  | 8.79 | 15.82  16.82  19.04 | Desister | 3 | 7 | | + |
|  | 6.19 | 14.22 | Desister | 7 | 7 | | + |
|  | 4.69 | 14.25  14.46 | Desister | 0 | 7 | | - |
|  | 10.38 | 17.32 | Desister | 0 | 0 | | - |
|  | 7.00 | 11.14  14.21 | Desister | 0 | 0 | | + |
|  | 7.19 | 14.68 | Desister | 0 | 0 | | + |
|  | 12.37 | 18.84 | Desister | 1 | 0 | | - |
|  | 10.70 | 17.09  19.38 | Persister | 6 | 6 | | - |
|  | 6.46 | 14.34 | Desister | 0 | 0 | | + |
|  | 5.85 | 13.31  17.65  35.14 | Persister | 6 | 6 | | + |
|  | 5.14 | 15.83 | Desister | 0 | 7 | | + |
|  | 11.13 | 16.88  18.28  23.53 | Desister | 6 | 6 | | - |
|  | 10.49 | 16.03  19.08 | Persister | 6 | 6 | | - |
|  | 5.34 | 20.00 | Desister | 6 | 6 | | + |
|  | 8.58 | 15.32 | Desister | 0 | 7 | | + |
|  | 11.07 | 16.74 | Desister | 3 | 4 | | + |
|  | 8.46 | 14.76 | Desister | 0 | 7 | | + |
|  | 5.61 | 14.96 | Desister | 0 | 7 | | - |
|  | 9.14 | 16.53 | Desister | 0 | 7 | | - |
|  | 3.75 | 13.09  19.65 | Desister | 5 | 3 | | + |
|  | 9.31 | 14.06 | Desister | 2 | 7 | | + |
|  | 12.29 | 15.06 | Desister | 3 | 7 | | - |
|  | 7.18 | 26.04 | Persister | 6 | 6 | | - |
|  | 11.01 | 17.95 | Desister | 0 | 7 | | - |
|  | 10.23 | 14.53 | Desister | 0 | 7 | | - |
|  | 8.58 | 15.01 | Desister | 0 | 0 | | + |
|  | 6.90 | 27.13 | Desister | 6 | 6 | | + |
|  | 10.71 | 14.63 | Desister | 5 | 7 | | - |
|  | 8.99 | 15.47 | Persister | 5 | 6 | | + |
|  | 3.77 | 27.51 | Desister | 5 | 5 | | + |
|  | 11.99 | 14.82 | Desister | 7 | 7 | | - |
|  | 9.68 | 16.03 | Desister | 0 | 0 | | - |
|  | 6.35 | 29.60 | Desister | 6 | 7 | | + |
|  | 5.59 | 22.53 | Desister | 6 | 6 | | + |
|  | 7.29 | 30.31 | Desister | 6 | 6 | | + |
|  | 4.72 | 27.29 | Desister | 6 | 6 | | + |
|  | 6.75 | 18.10 | Desister | 6 | 6 | | + |
|  | 8.22 | 29.48 | Persister | 6 | 6 | | + |
|  | 3.52 | 16.01 | Desister | 6 | 6 | | + |
|  | 5.91 | 20.09 | Desister | 4 | 6 | | + |
|  | 4.04 | 26.03 | Desister | 6 | 7 | | + |
|  | 6.94 | 28.70 | Desister | 5 | 6 | | + |
|  | 4.96 | 26.67 | Desister | 6 | 6 | | - |
|  | 5.09 | 26.59 | Desister | 6 | 6 | | + |
|  | 4.15 | 26.08 | Desister | 1 | 0 | | + |
|  | 5.22 | 26.12 | Desister | 5 | 6 | | + |
|  | 6.33 | 26.24 | Desister | 6 | 6 | | + |
|  | 3.95 | 23.60 | Desister | 5 | 6 | | + |
|  | 6.73 | 26.08 | Desister | 6 | 6 | | + |
|  | 3.85 | 16.26 | Desister | 0 | 7 | | + |
|  | 5.13 | 18.70 | Desister | 6 | 6 | | + |
|  | 5.25 | 24.24 | Desister | 6 | 6 | | + |
|  | 4.67 | 23.50 | Desister | 3 | 4 | | - |
|  | 7.13 | 14.58  20.35 | Desister | 6 | 6 | | + |
|  | 9.03 | 27.35 | Desister | 6 | 6 | | - |
|  | 5.19 | 16.29 | Desister | 6 | 1 | | + |
|  | 4.16 | 22.93 | Desister | 5 | 4 | | + |
|  | 8.11 | 16.63 | Desister | 2 | 7 | | - |
|  | 4.41 | 23.38 | Desister | 0 | 0 | | + |
|  | 4.32 | 21.65 | Desister | 6 | 6 | | + |
|  | 3.57 | 22.16 | Desister | 0 | 0 | | - |
|  | 6.58 | 17.11 | Desister | 6 | 6 | | - |
|  | 6.95 | 22.87 | Desister | 6 | 6 | | + |
|  | 3.98 | 16.97 | Desister | 0 | 0 | | + |
|  | 6.82 | 21.98 | Desister | 5 | 6 | | + |
|  | 6.02 | 17.06 | Desister | 0 | 0 | | + |
|  | 12.67 | 15.50 | Desister | 7 | 7 | | - |
|  | 4.06 | 16.84 | Desister | 6 | 6 | | + |
|  | 7.45 | 22.32 | Persister | 2 | 0 | | + |
|  | 9.46 | 14.35  14.94  18.47 | Desister | 0 | 0 | | - |
|  | 9.47 | 15.46 | Desister | 0 | 0 | | - |
|  | 10.30 | 20.27 | Desister | 6 | 6 | | + |
|  | 8.87 | 13.62 | Persister | 6 | 6 | | + |
|  | 5.83 | 17.14 | Desister | 3 | 3 | | + |
|  | 4.31 | 15.53 | Desister | 5 | 7 | | + |
|  | 9.27 | 20.75 | Desister | 0 | 0 | | - |
|  | 7.62 | 19.77 | Persister | 6 | 6 | | + |
|  | 8.19 | 19.05 | Desister | 5 | 0 | | - |
|  | 8.66 | 20.05 | Desister | 3 | 0 | | + |
|  | 12.82 | 16.90 | Persister | 0 | 7 | | - |
|  | 9.07 | 20.25 | Desister | 0 | 0 | | - |
|  | 5.35 | 17.43 | Desister | 6 | 6 | | + |
|  | 9.46 | 16.20 | Desister | – | – | | + |
|  | 10.35 | 21.18 | Persister | 6 | 6 | | + |
|  | 4.39 | 15.42 | Desister | 0 | 0 | | + |
|  | 3.69 | 15.09 | Desister | 4 | 0 | | + |
|  | 10.0 | 19.19 | Desister | 0 | 0 | | + |
|  | 4.53 | 14.51 | Desister | 0 | 7 | | + |
|  | 9.13 | 17.68 | Persister | 6 | 6 | | + |
|  | 6.39 | 16.23 | Desister | 1 | 0 | | + |
|  | 12.48 | 20.42 | Desister | 3 | 3 | | - |
|  | 8.84 | 16.04 | Desister | 3 | 3 | | + |
|  | 10.76 | 16.61 | Persister | 6 | 6 | | + |
|  | 12.99 | 20.22 | Desister | 0 | 7 | | - |
|  | 8.51 | 13.07 | Desister | 0 | 0 | | + |
|  | 11.53 | 18.96 | Desister | 5 | 6 | | + |
|  | 11.60 | 15.55  15.97 | Persister | 6 | 7 | | - |
|  | 9.68 | 15.69 | Persister | 6 | 6 | | + |
|  | 12.84 | 17.18 | Desister | 0 | 0 | | - |
| 111 | 3.75 | 31.28 | Desister | – | | – | + |
| 112 | 8.36 | 18.82 | Persister | (6) | | – | + |
| 113 | 8.69 | 33.34 | Desister | (6) | | – | - |
| 114 | 5.48 | 28.73 | Desister | (6) | | – | - |
| 115 | 4.63 | 27.73 | Desister | – | | – | + |
| 116 | 7.30 | 16.67 | Desister | (6) | | – | - |
| 117 | 5.39 | 27.75 | Desister | (6) | | – | - |
| 118 | 5.88 | 28.11 | Desister | (6) | | – | - |
| 119 | 9.27 | 31.40 | Desister | (6) | | – | - |
| 120 | 7.82 | 29.57 | Desister | – | | – | - |
| 121 | 6.93 | 28.69 | Desister | – | | – | - |
| 122 | 3.33 | 24.77 | Desister | (6) | | – | + |
| 123 | 4.65 | 24.77 | Desister | (0) | | – | + |
| 124 | 6.16 | 25.60 | Desister | (0) | | – | + |
| 125 | 6.35 | 25.23 | Desister | – | | – | - |
| 126 | 5.28 | 23.65 | Desister | (6) | | – | + |
| 127 | 4.73 | 23.50 | Desister | (0) | | – | + |
| 128 | 11.14 | 21.58 | Desister | (6) | | – | - |
| 129 | 3.84 | 22.47 | Desister | (6) | | – | + |
| 130 | 11.24 | 23.16 | Desister | (6) | | – | - |
| 131 | 4.60 | 24.18 | Desister | (6) | | – | + |
| 132 | 10.21 | 28.15 | Desister | – | | – | - |
| 133 | 5.55 | 18.10 | Desister | (6) | | – | + |
| 134 | 10.18 | 21.55 | Desister | (6) | | – | + |
| 135 | 12.06 | 23.98 | Desister | (6) | | – | + |
| 136 | 6.76 | 19.50 | Desister | – | | – | + |
| 137 | 11.91 | 23.29 | Desister | – | | – | - |
| 138 | 8.79 | 18.19 | Desister | – | | – | + |
| 139 | 11.90 | 21.17 | Desister | (6) | | – | - |

| *Note.* For Kinsey ratings (last 12 months), 0 = exclusively gynephilic in relation to birth sex and 6 = exclusively androphilic in relation to birth sex. A 7 = no reported sexual fantasies or sexual behavior. A dash in the Kinsey columns indicate that data were not available. A bracketed score in the Kinsey Fantasy column indicates that the participant did not complete a full sexual orientation interview but sufficient data were available to inform a Kinsey rating. In the DSM column, a plus sign indicates the participant meet full *DSM-III, DSM-III-R,* or *DSM-IV* criteria for GID at the childhood assessment. A minus sign indicates the participant was subthreshold for the diagnosis of GID.    ^a^Some participants were assessed at more than one follow-up points. In these cases, data from the most recent assessment were used. |
| --- |
