# Supplementary material for: A Follow-Up Study of Boys With Gender Identity Disorder
Source: Front Psychiatry. 2021 Mar 29;12:632784. doi: 10.3389/fpsyt.2021.632784 (PMC8039393; doi:10.3389/fpsyt.2021.632784)
Supplement: Supplementary file 5 [file Data_Sheet_3.docx]

**Supplemental Text**

There were no significant group differences on the demographic variables of age, social class, and ethnicity. An analysis of variance (ANOVA) on childhood IQ approached significance, *F*(2, 162) = 2.99, *p* = .053. Duncan’s multiple range test for unequal Ns showed that the participants did not differ significantly from the other two groups; however, the non-participants who declined to participate had a higher IQ in childhood than those who could not be traced. Regarding parent-report of behavior problems on the three CBCL indices, there were no significant differences between the three groups. There were also no significant group differences on any of the measures of sex-typed behavior.

**Supplemental Table 1**

There was notable variation within the group of persisters in the extent to which they had socially and medically transitioned to the female gender role (see Supplemental Table 1). At follow-up, the majority of the gender dysphoric participants (*n* = 9) were not receiving any biomedical treatments to either suppress physical development of secondary sex characteristics/masculinization of their body or to feminize their body. The remaining 8 (47.1%) participants were taking puberty suppressing hormones or were on cross-sex hormonal therapy to feminize their physical appearance. Of the 9 participants who were not taking puberty suppressing or cross-sex hormones, 6 did not report any attempt to present socially in the female gender role (e.g., using a female name, living in the female role). Of the remaining 3 participants, 2 were attempting to live socially in the female gender role and data were incomplete for 1 participant. None of the 17 gender dysphoric participants had received any type of surgery to feminize their appearance (e.g., breast implants, facial feminization surgery, genital reconstruction).

Among the 17 persisters, 11 were using a female name. Of these 11 participants, 2 had legally changed their name on official documents (e.g., driver’s license) and 1 was in the process of pursuing a legal name change. Nine of the gender dysphoric participants were living in the female gender role and 4 of them were living in the male gender role. Of the remaining 4 participants, 1 was living partially in the female role (e.g., would sometimes wear make-up and at others times presented androgynously, but did not wear stereotypic female clothing), 2 presented androgynously, and data were not available for 1 participant.

None of the 122 participants classified as desisters desired cross-sex hormones or sex reassignment surgery to feminize their bodies nor did they express a desire to get rid of their male sex characteristics. None of the desisters presented socially as women nor did they express the desire to socially transition to the female gender role (e.g., name change, clothing choice).

**Supplemental Table 2**

Between-Groups Analyses (Individual Measures) for Supplemental Table 2

The ANCOVA for the cross-sex toy preference on the Playmate and Play Style Preferences Structured Interview approached significance (*p* = .096). The significant one-way ANCOVAs were followed up with post hoc analyses using lmatrix commands and the significant chi-square was followed-up with pair-wise comparisons.

On the Draw-a-Person, there was one significant post-hoc contrast. The biphilic/ androphilic persisters were, on average, significantly more likely to draw a female first compared to the gynephilic desisters (*p* = .04). The comparison between the biphilic/ androphilic desisters and gynephilic desisters approached significance (*p* = .09), with the biphilic/androphilic desisters showing a greater tendency to draw a female first. The comparison between the biphilic/ androphilic persisters and biphilic/androphilic desisters was not significant. On the free play measure, all post-hoc contrasts were significant. The biphilic/ androphilic persisters had, on average, more cross-sex play than did the biphilic/androphilic desisters and the gynephilic desisters. The latter two groups differed significantly from each other; the biphilic/androphilic desisters had, on average, significantly more cross-sex play than did the gynephilic desisters. On the Gender Identity Interview, the biphilic/androphilic persisters reported, on average, significantly more gender confusion than did the biphilic/ androphilic desisters. The post-hoc comparison between the biphilic/androphilic persisters and the gynephilic desisters approached significance (*p* = .054), with the biphilic/androphilic persisters reporting more gender confusion than the gynephilic desisters. The comparison between the biphilic/androphilic desisters and gynephilic desisters was not significant. There was also a significant group difference on the peer preference domain of the Playmate and Play Style Preferences Structured Interview. The biphilic/androphilic persisters had, on average, significantly more cross-sex peer preference compared to the biphilic/androphilic desisters and the gynephilic desisters, who did not differ significantly from each other. On the Gender Identity Questionnaire for Children, a parent-report measure of cross-gender interest and identification, the biphilic/androphilic persisters were reported to be significantly more cross-gendered than the biphilic/androphilic desisters and the gynephilic desisters. The latter two groups did not differ significantly from each other.
